# The danish regions pediatric triage model has a limited ability to detect both critically ill children as well as children to be sent home without treatment – a study of diagnostic accuracy

**DOI:** 10.1186/s13049-017-0397-6

**Published:** 2017-05-30

**Authors:** Lotte Høeg Hansen, Christian Backer Mogensen, Lena Wittenhoff, Helene Skjøt-Arkil

**Affiliations:** 1 The Family Center, Hospital of Southern Jutland, Kresten Philipsensvej 15, 6200 Aabenraa, Denmark; 2 The Emergency Department, Hospital of Southern Jutland, Kresten Philipsensvej 15, 6200 Aabenraa, Denmark; 3 Focused Research Unit in Emergency Medicine, Institute of Regional Health Research, University of Southern Denmark, J.B.Winsløws Vej 19, 5000 Odense C, Denmark

**Keywords:** Pediatric, Triage, Reference standard, The Danish Regions Pediatric Triage model

## Abstract

**Background:**

The Danish Regions Pediatric Triage model (DRPT) was introduced in 2012 and subsequent implemented in most Danish acute pediatric departments. The aim was to evaluate the validity of DRPT as a screening tool to detect both the most serious acute conditions and the non-serious conditions in the acute referred patients in a pediatric department.

**Method:**

The study was prospective observational, with follow-up on all children with acute referral to pediatric department from October to December 2015. The DRPT was evaluated by comparison to a predefined reference standard and to the actual clinical outcomes: critically ill children and children returned to home without any treatment. The sensitivity, specificity, positive predictive value, negative predictive value, accuracy and likelihood for positive and negative test were calculated.

**Results:**

Five hundred fifty children were included. The DRPT categorized 7% very urgent, 28% urgent, 29% standard and 36% non-urgent. The DRPT was equal to the reference standard in 31% of the children (CI: 27-35%). DRPT undertriaged 55% of the children (CI: 51-59%) and overtriaged 14% of the children (CI: 11-17%). For the most urgent patients the sensitivity of DRPT was 31% (CI: 20-48%) compared to the reference standard and 20% (CI: 7-41) for critically ill. For children with non-urgent conditions the specificity of DRPT was 66% (CI: 62-71%) compared to the reference standard and 68% (CI: 62-75%) for the children who went home with no treatment. In none of the analyses, the likelihood ratio of the negative test was less than 0.7 and the positive likelihood ratio only reached more than 5 in one of the analyses.

**Discussion:**

This study is the first to evaluate the DRPT triage system. From the very limited validity studies of other well-established triage systems, it is difficult to judge whether the DRPT performs better or worse than the alternatives. The DRPT errs to the undertriage side. If the sensitivity is low, a number of the sickest children are undetected and this is a matter of concern.

**Conclusion:**

The DRPT is a triage tool with limited ability to detect the critically ill children as well as the children who can be returned to home without any treatment.

**Trial registration:**

Not relevant

**Electronic supplementary material:**

The online version of this article (doi:10.1186/s13049-017-0397-6) contains supplementary material, which is available to authorized users.

## Background

Generally, the pediatric acute visitation sections become larger and a higher number of children are waiting for a longer time. It is therefore necessary to prioritize which child is most critically ill, and should be seen by the physician first [1–5]. However, the serious condition of critically ill children is not always recognized, when the children enter the pediatric department, even when assessed by experienced pediatric nurses [3, 5, 6]. This has led to the introduction of triage as a method for the nurses to determine the order in which the patients are seen by the physician. Therefore, triage should help the nurse differentiate the critically ill patients who need immediate attention, from patients who can safely wait.

However, there are some challenges to triage. Over-triage leads to unnecessary use of health professional resources, while under-triage results in unrecognized critically ill children [2, 4, 5, 7, 8]. Triage models use various indicators of urgency – for example some are based on primary complaints or symptoms, some on expected use of resources, and some on vital parameters [1, 4, 9]. If the triage is based on vital signs, it is influenced by a range of factors such as fever, anxiety and crying. Furthermore, there is not complete consensus on reference values for vital parameters measured in children at various ages [10–12].

It is thus paramount to evaluate, whether or not a triage system can predict the true urgency of the child [4]. True urgency is, however, a parameter that is very difficult to measure. Therefore, other indicators of urgency (proxy variables) are necessary in order to make an estimate. Two methods have been used in evaluation of pediatric triage models. One method uses a construction of a reference standard for the urgency, based on literature review and expert opinions [5]. The other method uses clinical outcomes, like admission to hospital or to intensive care unit, returned to home and utilized resources, as indicators for the urgency [2, 9, 13, 14]. The four most commonly used triage systems in pediatrics are the Manchester Triage System (MTS), the Emergency Severity Index (ESI), the Canadian Triage and Acuity Scale (CTAS) and the Australian Triage scale (ATS) [4, 6]. They have all been evaluated by one of these methods [2, 4, 5, 9].

The Danish Regions Pediatric Triage model (DRPT) was introduced in 2012 and has now been implemented with some local modifications in most Danish acute Pediatric Departments. The DRPT has not been validated so far. We thus aimed to evaluate the validity of DRPT as a screening tool to detect both the most serious acute conditions and the non-serious conditions in daily clinical use in a Pediatric Department.

## Methods

### Study design and setting

We conducted a prospective observational study, made as a cohort study with follow-up. The study was designed and reported to conform to STARD (Standards for Reporting of Diagnostic Accuracy Studies) guidelines [15]. The study was run through the Research Unit in Emergency Medicine at the University of Southern Denmark, Institute of Regional Research, Southern Center.

### Study population

The hospital of Southern Denmark serves a population of 250.000 citizens, and the attendance to the pediatric department is approximately 3.000 children per year. Most of the children attending the acute pediatric visitation section (APVS) are referred by general practitioners A minority is brought by ambulance without previous medical evaluation. We consecutively included all children with acute referral to our Pediatric Department from October to December 2015. Patients younger than 28 days were received at the neonatal ward and not included in the study. We made a pamphlet with brief information about the study and a formula for the parent to sign as their consent. We included both children referred with suspected medical and with surgical diagnosis.

### Study instrument

DRPT is a 4-level triage system based on vital signs (pulse, respiration rate, arterial oxygen saturation, Glasgow Coma Scale, temperature) with age specific cut-off values combined with a presenting complaint algorithm. The presenting complaint algorithm consists of the main presenting complaint, CNS symptoms and symptoms of respiration. The chosen triage level is determined by the variable that indicates the highest degree of urgency. For further description, see Additional file 1.

When the DRPT was implemented at our Pediatric Department, it was decided to add a subgroup to the reference values of heart rate and respiratory frequency for 3-5 month old children. This was the only difference between our triage model and the original DRPT model.

All children were supposed to be triaged by a trained nurse within 10 min after arrival at the APVS. The “not urgent” patient could safely wait, and was re-triaged every 2 h until examined by a doctor. The “standard” patient waited maximum 90 min, and was re-triaged every 30 min until examined by a doctor. The “urgent” patient waited for maximum 15 min. At the arrival of a “very urgent” patient, the doctor was called to the room immediately.

On arrival to the APVS, a triage nurse obtained the information concerning main complaints, assessed the child, measured the vital values and determined the triage level based on these findings. All the information was documented on a standardized paper form as part of the patient file.

### Data sources and collection

The patient files were reviewed by two of the authors to obtain the triage information and the defined outcomes. The following variables were registered: heart rate, arrhythmia, respiratory frequency and pattern, saturation, Glascow Coma Scale, capillary response, temperature, relevant objective findings and relevant symptoms, presence of predefined possible life-threatening conditions, x-ray, CT and ultrasound investigations, ECG and cardiac monitoring, all treatments including inhalation therapy, IV fluid and medication, oral medication, referral to specialist and whether the child was returned to home, admitted or required intensive care. The data was registered using a predesigned electronic study form drafted in the surveying tool SurveyXact ®.

For the present study, we used the “ideal” triage level, which is defined as the triage level obtained if the triage process was consistently executed according to the DRPT protocol.

Only the clinical information was available to the two data collectors. The DRPT and reference standard triage were calculated afterwards based on the clinical data.

### Reference standards

We applied a formerly used proxy-method and the actual clinical outcomes to determine the true urgency.

As a formerly used reference standard we replicated the method developed by van Veen et al [5]. In short it has 5 urgency levels: immediate, very urgent, urgent, standard and not urgent (see Table 1). The most urgent level includes patients with vital parameters, which are abnormal in referral to the reference standards of the pediatric risk of mortality score (PRISM III) [16]. The second level of urgency is a specified range of life threatening conditions, but not fulfilling the criteria for the most urgent level. The third and fourth categories were defined by a number and combinations of diagnostic investigations, interventions and follow-up. The fifth category did not require any of the above [5]. Since the DRPT model has only four levels (very urgent, urgent, standard, not-urgent) we merged the immediate and very urgent level in the reference model into one category.

**Table 1:** Reference standard definitions

Urgency levels	Definition of content
Immediate:	Abnormal age adjusted vital parameters according to PRISM III^a^
Very urgent:	Presence of a possible life threatening condition: Meningitis, severe sepsis, high-energy trauma, substantial external blood loss or trauma (sharp/blunt) leading to substantial blood loss, aorta dissection, .10% dehydration, (near) drowning, electric trauma, apparently life-threatening event (ALTE), possible dangerous intoxication, .10% burns, facial burns or possible inhalation trauma.
Urgent:	Specific combinations of diagnostic work-up, therapy and follow-up.
Standard:	Other combinations of diagnostic work-up, therapy and follow-up
Non-urgent:	No diagnostic work-up or follow-up. Therapy restricted to simple advice and/or medicine on prescription, or none at all.

Adopted from van Veen et al. 2008. In our study we merged "Immediate" and "very urgent"

^a^PRISM III: pediatric risk of mortality score III

As actual clinical outcomes we used 1) presence of possible life threatening condition or transferring to a higher hospital or ICU as critically ill patients and 2) no admission as patients who went home with no treatment.

### Equipment

For the measurement of temperature, we used a Braun Welch Allyn ear thermometer and for the measurement of pulse, blood pressure, saturation and respiratory frequency a Philips Intelli Vue MP 50.

### Sample size

In former studies of triage the most urgent triage level (immediate + very urgent) accounted for approximately 20% of the children [2, 5]. In order to obtain 100 children at this triage level we aimed to include a minimum of 500 children.

### Data analysis

Data was transferred to and analysed in STATA14. The triage of DRPT and standard reference were calculated according to the existing collected clinical data. Missing data were viewed as not relevant for the triage assessment. We calculated the triage distribution of DRPT and the over- and undertriage compared with the reference standard including 95% confidence intervals. The DRPT was then analysed as a screening tool, as suggested by Hardern [17]. We made four analyses: With the reference standard as the gold standard using the same methodology as proposed by van Veen et al [5] we analysed (I) the DRPT ability to detect the children with the most urgent needs. The DRPT and reference standard was dichotomized to a very urgent triage level versus the urgent + standard + non-urgent level. To evaluate (II) the ability of the DRPT model to detect children who had no urgent needs according to the reference model. The DRPT and the reference standard were dichotomized to non-urgent level versus the very urgent + urgent + standard level. We continued using the actual clinical outcome as gold standard and (III) analysed the DRPT model as a screening tool to detect the children who actually had a life threatening condition, including those children who were transferred to the intensive care unit. Again, we dichotomized the DRPT in the very urgent triage level versus the other levels. Finally, we (IV) analysed the ability of the DRPT model to detect children who were returned to home without any treatment, by dichotomizing the DRPT in the non-urgent level against the other levels.

For all these analysis, we calculated the sensitivity, specificity, positive predictive value, negative predictive value, accuracy and likelihood for positive and negative test, all with a 95% confidence interval [17].

### Ethical approval

The regional ethical committee of Southern Denmark waived the need for approval as the study was considered a quality assurance project. We obtained parental written consent to participate and retrieve information in the patient file. The study was registered with the Danish Data Protection Agency (15/39212-1691).

## Results

**Fig. 1:**
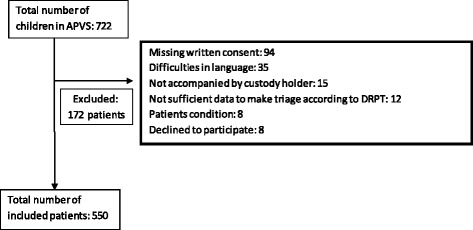
Distribution of the patients

We included 550 out of 722 children, who were seen in the APVS (Fig. 1). We excluded 172 children of which 75% was due to missing written consent and difficulties in language. Eight children were excluded because they were transferred directly to a higher hospital level, or were so ill, that it was considered inappropriate to ask for inclusion in the study.

The median age of the children was 39 months (Inter Quartile Range 14-128 months) and 50% of the children were boys. The age distribution and reasons for referral to the APVS is shown in Tables 2 and 3. Among the children, 36% were admitted to the hospital, the remaining returned to home from the APVS.

**Table 2:** Age distribution

Age	n(%)
<3 months	33(6%)
3-6 months	15(3%)
6-12 months	62 (11%)
1-3 years	153 (28%)
3-8 years	115(21%)
>8 years	172(31%)

**Table 3:** Distribution of reasons for referral (1)

Neurological	65 (11%)
Persisting seizures	23 (4%)
Other neurological symptoms	18(3%)
Head ache	14 (2%)
Ended seizures	10(2%)
Respiratory	148 (26%)
Other airway problems	69 (12%)
Astmatic bronchitis	31(5%)
Croup	28(5%)
Pneumonia	20(3%)
Abdominal and urological	148 (26%)
Stomach ache	54 (9%)
Appendicitis	31(5%)
Other gastrointestinal symptoms	23 (4%)
Gastroenteritis	21(4%)
Urine tract infection	19(3%)
Fever	68(12%)
Other reasons	151(26%)
Others	43(7%)
Rashes	24(4%)
Dehydration	22(4%)
Pain	22(4%)
Poisoning	20(3%)
Other infections	20(3%)

(1) some patients have more than one reason

The DRPT categorized 7% of the children to be very urgent, 28% urgent, 29% standard and 36% non-urgent (Table 4).

**Table 4:**
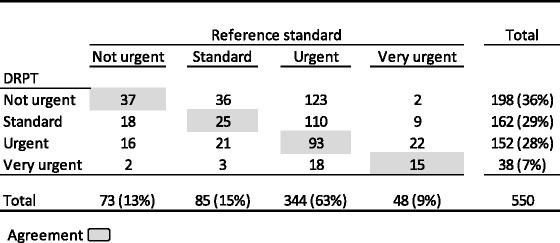
Distribution of triage levels in DRPT and the reference standard

The DRPT agreed with the reference standard in 170 patients (31%; 95%-CI:27-35%). DRPT undertriaged 302 of the children (55%; 95%-CI: 51-59%) and overtriaged 78 patients (14%, 95%-CI: 11-17%) (Table 5A). Table 5B shows the correlation between reasons for referral and under- and overtriage. The DRPT under-performs mainly in patients with respiratory (52%), abdominal/urological (68%) and other (55%) reasons for referral. The patients with neurological (20%) and respiratory (16%) reasons for referral are those who are overtriaged the most.

**Table 5:** Comparing DRPT and the reference standard according to agreement on triage, undertriage and overtriage divided by A) urgency levels and B) reasons for referral

A			
Urgency levels	Agree	Undertriage	Overtriage
Not urgent	37	161	0
Standard	25	119	18
Urgent	93	22	37
Very urgent	15	0	23
Total	170 (31%)	302 (55%)	78 (14%)
B			
Reasons for referral	Agree	Undertriage	Overtriage
Neurological	40%	40%	20%
Respiratory	32%	52%	16%
Abdo mi nal/urological	20%	68%	11%
Fever	42%	44%	13%
Other	33%	55%	11%

The analysis of the DRPTs ability to identify the very urgent, the non-urgent, the most critically ill patients and the patients who could be returned to home without any further treatment is shown in Table 6.

**Table 6:** The Danish Regions Pediatric triage as a screening tool to detect the most urgent and non-urgent children

	Gold standard			
	Standard reference		Clinical outcome	
	I	II	III	IV
	Ability to detect very urgent patients	Ability to detect the no-urgent patients	Ability to detect critically ill patients (1)	Ability to detect patients who went home with no treatment
Number of patients				
True positive	15	37	5	132
True negative	479	316	492	143
False positive	23	161	33	66
False negative	33	36	20	209
Screening values				
Sensitivity	31% (19-46)	51% (39-63)	20% (7-41)	39% (34-44)
Specificity	95% (93-97)	66% (62-71)	94% (91-96)	68% (62-75)
Positive predictive value	40% (24-57)	19% (14-25)	13% (4-28)	67% (60-73)
Negative predictive value	94% (91-96)	90% (86-93)	96% (94-98)	41% (36-46)
Accuracy	90% (87-92)	64% (60-68)	90% (88-93)	50% (46-54)
Likelihood ratios				
of positive test	6.8 (3.8-12.2)	1.5 (1.2-2.0)	3.2 (1.4-7.5)	1.2 (0.9-1.6)
of negative test	a7 (a6-0.9)	a7 (0.6-a9)	0.9 (0.7-1.0)	ag (a8-1.0)

95%-confidence intervals are indicated in brackets

(1) Critical ill is defined as transferring to a higher hospital level or ICU, assigned by nurse during review of patient file, dangerous intoxication, severe sepsis, foreign body, critical hyperglycemia, pertussis, seizures, acute peritonsillar abscess and respiratory insufficiency

For the most urgent patients the sensitivity of DRPT was 31% (95%-CI: 20-48%) compared to reference standard and 20% (95%-CI: 7-41) when critically ill outcome was used as reference. For those children who were not urgent the specificity of DRPT was 66% (95%-CI: 62-71%) compared to the reference standard and 68% (95%-CI: 62-75%) when the children who went home with no treatment was used as reference. In none of the four analyses, the likelihood ratio of the negative test was less than 0.7 and the positive likelihood ratio only reached more than 5 in one of the analyses.

The analysis of the DRPTs ability to identify the most critically ill patients and the patients who could be returned to home without any further treatment divided by reasons of referral is shown in Table 7.

**Table 7:** The Danish Regions Pediatric triage as a screening tool to detect A) the most ill children and B) those who went home with no treatment. Both divided by reasons for referral

	Neurological reasons for referral	Respiratory reasons for referral	Abdominal and urological reasons for referral	Fever as reason for referral	Other reasons for referral
A					
Ability to detect critically ill patients					
Number of patients					
True positive	1	1	0	0	3
True negative	60	125	143	58	131
False positive	3	19	3	7	5
False negative	1	3	2	3	12
Screening values					
Sensitivity	50% (1-99)	25% (1-81)	0% (0-84)	0% (0-71)	20% (4-48)
Specificity	95% (87-99)	87% (80-92)	98% (94-100)	89% (79-96)	96% (92-99)
PPV	25% (1-81)	5% (0-25)	0% (0-71)	0% (0-41)	38% (9-76)
NPV	98% (91-100)	98% (93-100)	99% (95-100)	95% (86-99)	92% (86-96)
Accuracy	94% (88-100)	85% (79-91)	97% (94-100)	85% (77-94)	89% (84-94)
Likelihood ratios					
of positive test	10.5 (1.8-61.8)	1.9 (0.3-10.9)	0.0	0.0	5.4 (1.4-20.5)
of negative test	0.5 (0.1-2.1)	0.9 (0.5-1.5)	1.0 (1.0-1.1)	1.1 (1.0-1.2)	0.8 (0.6-1.1)
B					
Ability to detect patients who went home with no treatment					
Number of patients					
True positive	11	30	40	6	50
True negative	22	34	37	26	36
False positive	28	75	39	33	44
False negative	4	9	32	3	21
Screening values					
Sensitivity	28% (15-45)	29% (20-38)	51% (39-62)	15% (6-31)	53% (43-64)
Specificity	85% (65-96)	79% (64-90)	54% (41-66)	90% (73-98)	63% (49-76)
PPV	73% (45-92)	77% (61-89)	56% (43-67)	67% (30-93)	70% (58-81)
NPV	44% (30-59)	31% (23-41)	49% (37-60)	44% (31-58)	45% (34-57)
Accuracy	51% (38-63)	43% (35-51)	52% (44-60)	47% (35-59)	
Likelihood ratios					
of positive test	1.8 (0.7-5.1)	1.4 (0.7-2.6)	1.1 (0.8-1.5)	1.5 (0.45.5)	1.4 (10-2.1)
of negative test	0.9 (0.7-1.1)	0.9 (0.7-1.1)	0.9 (0.7-1.3)	0.9 (0.8-11)	0.7 (0.6-1.0)

95%-confidence intervals are indicated in brackets

For the critically ill patients and the patients who went home with no treatment, the sensitivity of DRPT was 53% and below for all groups of reasons for referral when compared to reference standard. The specificity was highest (98%, 95%-CI: 94-100) for the analysis of patient who came to the hospital with abdominal and urological complains and was critically ill. Analyses of critically ill patients with neurological reasons of referral and patient with fever who went home with no treatment had sensitivity of 90% and above. Only in the analysis of neurological reasons for referral when critically ill outcome was used as reference, the likelihood ratio of the negative test was less than 0.7. The positive likelihood ratio only reached more than 5 in two of the analyses being neurological and other reasons for referral when critically ill outcome was used as reference.

## Discussion

We have shown that, according to a predefined standard, the DRPT resulted in a correct triage of only 31%, an undertriage of 55% and overtriage of 14%. Depending on the choice of gold standard, the DRTP as a screening measure to find the most urgent patient reached a sensitivity of only 20-31% and had a specificity to identify children who could be returned to home with no treatment of 66-68%. The likelihood ratios for positive and negative tests indicated that DRPT as a diagnostic test only changed the likelihood of the condition to a minimal degree. These findings suggest that the DRPT is a weak triage system to identify the few most ill children, who need immediate care as well as the many children who can be sent home with no further intervention.

This study is the first to evaluate the DRPT triage system and we are thus unable to compare our results directly to other studies of DRPT. We can compare, however, the DRPT triage to other triage systems, which has undergone the same type of validation. A range of studies of Canadian Triage and Acuity Scale has found a good correlation between triage level and a range of surrogate markers of acuity, but no under- or overtriage, sensitivities or specificities have been reported [18]. In the ESI, Bauman found good correlations between triage and resources allocated and hospitalisation [2] but Travers found among 1173 children undertriage (11%) and 16% overtriage for hospitalisation. The MTS has been more extensively evaluated by Roukema in 2008 against a reference classification, who found that undertriage occurred in 15% of patients and overtriage in 40%, and the sensitivity of the MTS to detect emergent/very urgent cases was 63% [19]. In 2008, Van Veen et al found, against the same reference classification as used in our study, that in a population of more than 17.000 children, the MTS agreed with the reference standard in 34%, while 54% children were overtriaged and 12% undertriaged, with a sensitivity of 63% and a specificity of 79% for identifying high urgency patients [5]. None of these studies has focused on patients, who could be safely sent home.

Compared with these results it seems that DRPT has a lower ability to detect the high urgency patients than MTS. No comparison can be made against the ESI and Canadian triage scale. While both the DRPT and the MTS only agree with the reference standard in 30-34%, the DRPT has a problem with undertriage in comparison with the MTS, with a sensitivity of only 20-31% where MTS has sensitivity of 63%.

Our results have some clinical implications. From the very limited validity studies of other well-established triage systems, it is difficult to judge whether the DRPT performs better or worse than the alternatives. While the MTS triage system also had a moderate validity, it errs on the safe side with a high degree of overtriage. The DRPT errs to the undertriage side. If the sensitivity is low, a number of the sickest children are undetected and this is a matter of concern. Furthermore, a specificity of around 65% for children who could be returned to home without treatment does not add much help for an early identification of this group of children.

The DRPT triage system has already been implemented in the Danish pediatric departments, and the results of our study encourage to awareness of the fact, that some of the children might suffer from more urgent conditions than the triage indicates. It also invites to further studies of the DRPT triage to assess, if the same results are seen at other sites, and to further analyse where the triage system can be improved to detect the sickest children better. Finally, we believe that no triage system will eliminate that assessing the condition of a child is difficult and often requires observation time and considerable experience. A recent study has shown that experienced pediatric triage nurses have significant higher prediction accuracy for pediatric admission than well-established triage and prediction tools [7]. Our results support that DRPT triage of children should always be accompanied by a clinical judgement from an experienced health professional and raises the question whether the DRPT adds any value to the clinical assessment.

More generally, the idea of evaluating a triage system has been questioned due to some inherent weaknesses in this evaluation. There is no well-defined gold standard for assessing urgency at presentation. The reference standard made by van Veen et al. is the only reference standard made for pediatric triage until now. However a gold standard based on utilized resources does not necessarily reflect the true triage urgency and accuracy, and it is depend on local organisation. Hospitalization, admission to ICU and utilization of resources are only surrogate markers of urgency [20] and does not take into account the dynamic nature of the patient condition and interventions, where the direction of the patient condition might change over short time. Finally, triage is a way of defining priority of care more than severity which might limit the evaluation a triage system value. While some authors argue that it is not possible to measure the effect of triage, others believe that convincing demonstrations of the influence of triage is possible [21].

### Strengths and limitations

The strength of the present study is that it is the first study to evaluate a triage system, which has already been implemented in most Danish pediatric departments. It uses two different methods in four validating models of the triage system and refers to a reference standard, which enables us to make a comparison with another well-established triage system. There are, however, some limitations to the study. It is small, as reflected in the wide confidence intervals around the results. It is a single-site study, which might limit the external validity. Some of the very urgent patients were not included in the study, because the nurses found it inappropriate to ask for consent. We also examined the ideal triage level based on the recorded information on the triage sheet, not the actual triage level. We did so, because we observed that quite often the triage nurses chose another triage level than a strict adherence to the DRPT protocol indicated. We are currently investigating, whether this was due to inexperience, lack of knowledge of the DRPT triage system or because the triage nurse overruled the DRPT triage.

## Conclusion

In conclusion, we found that the DRPT is a triage tool with limited ability to detect as well the sickest children, as the children, who can be returned to home. Especially the low sensitivity to detect the critically ill children is a matter of concern. We encourage further studies of the DRPT to assess, if our results are replicable elsewhere, and to analyse if modifications of the already implemented DRPT can improve the validity.

## Additional file

Additional file 1: The Danish Regions Pediatric Triage Model. (DOCX 368 kb)

## Abbreviations

APVS: Acute pediatric visitation section
ATS: Australian triage scale
CI: Confidence interval
CNS: Central nerve system
CRP: C-reactive protein
CT: Computer tomography
CTAS: Canadian Triage and acuity scale
DRPT: The Danish Regions Pediatric Triage model
ECG: Electrocardiography
ED: Emergency Department
ESI: Emergency Severity Index
ICU: Intensive Care Unit
MTS: Manchester Triage System
