# Supplementary material for: The danish regions pediatric triage model has a limited ability to detect both critically ill children as well as children to be sent home without treatment – a study of diagnostic accuracy
Source: Scand J Trauma Resusc Emerg Med. 2017 May 30;25:55. doi: 10.1186/s13049-017-0397-6 (PMC5450070; doi:10.1186/s13049-017-0397-6)
Supplement: Additional file 1: — The Danish Regions Pediatric Triage Model. (DOCX 368 kb) [file 13049_2017_397_MOESM1_ESM.docx]

**Additional file 1 - The Danish Regions Pediatric Triage Model**

The Danish Regions Pediatric Triage Model has been translated from the Danish version by the authors.

**
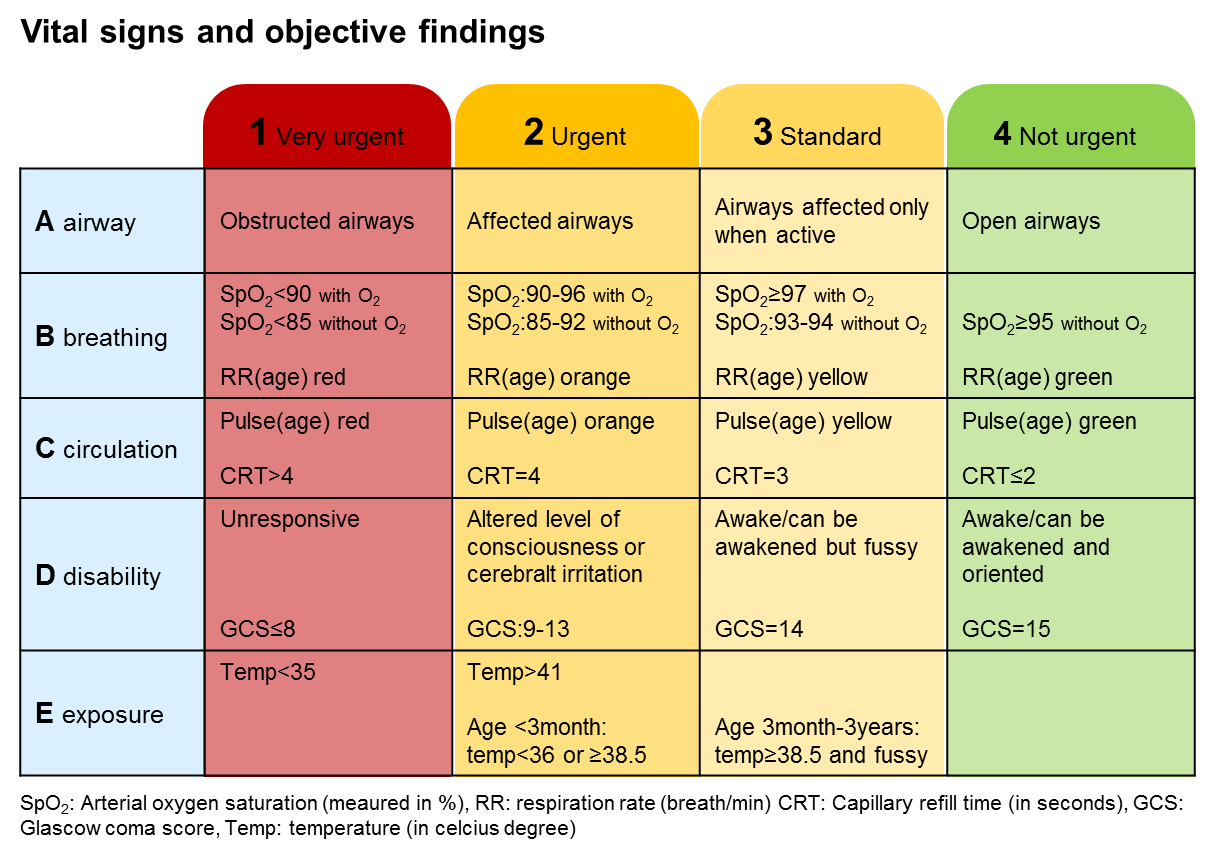
**

**
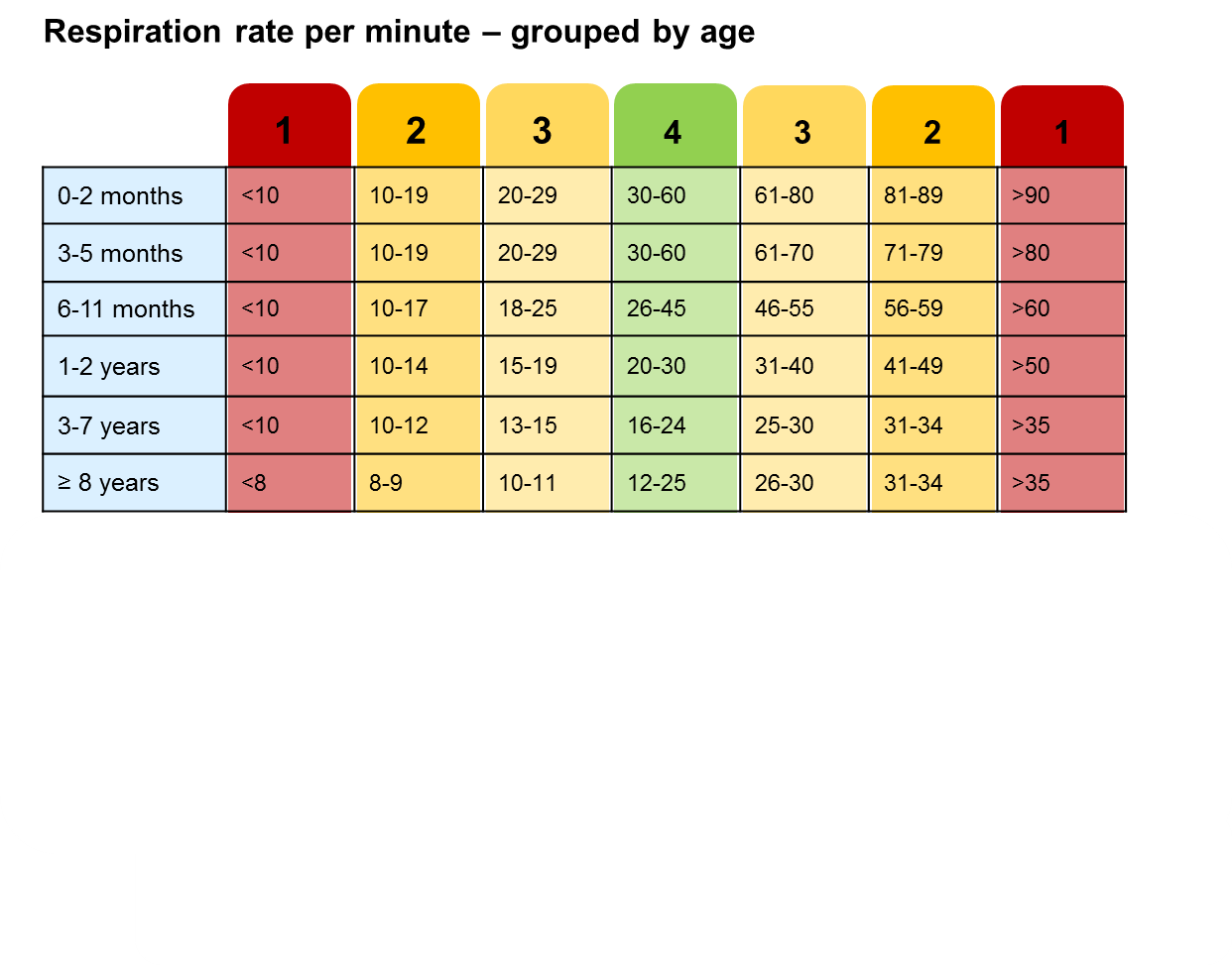
**

**
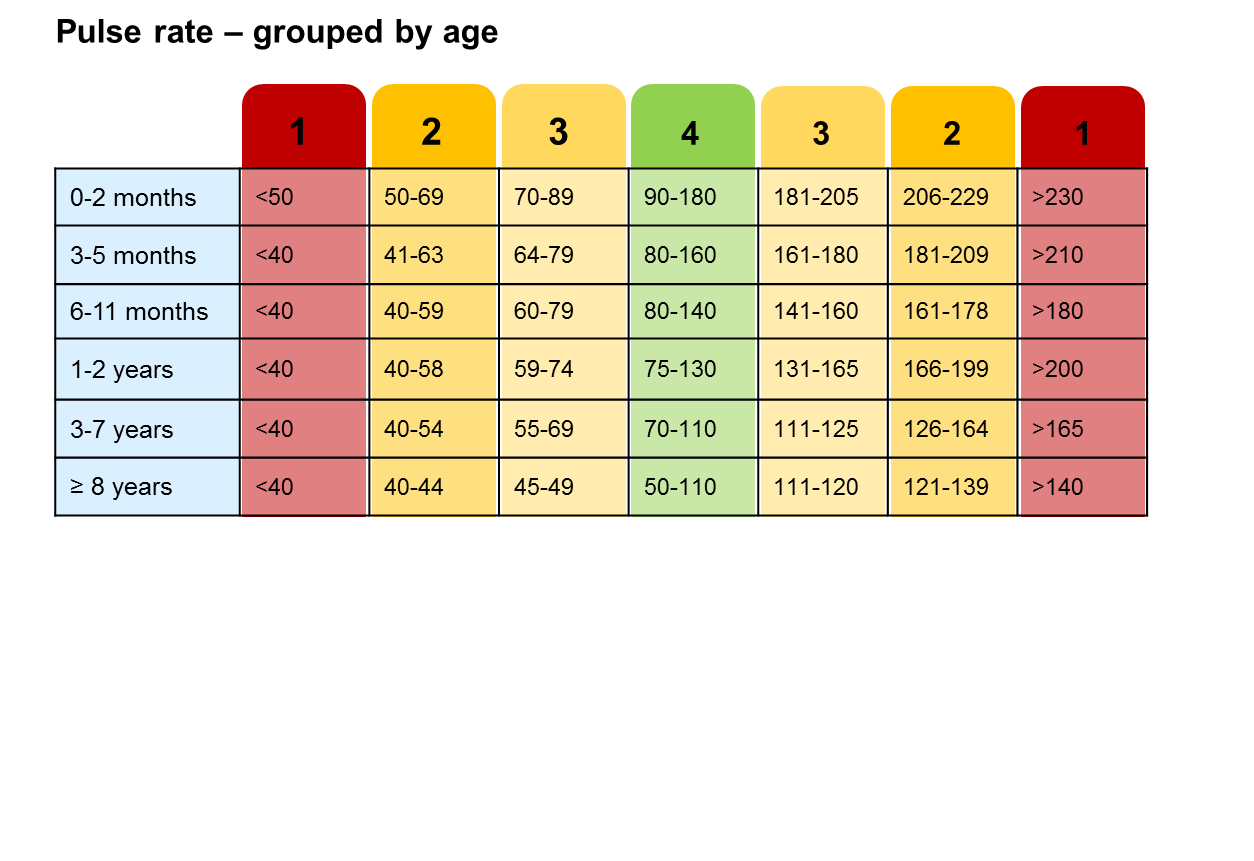
**

**
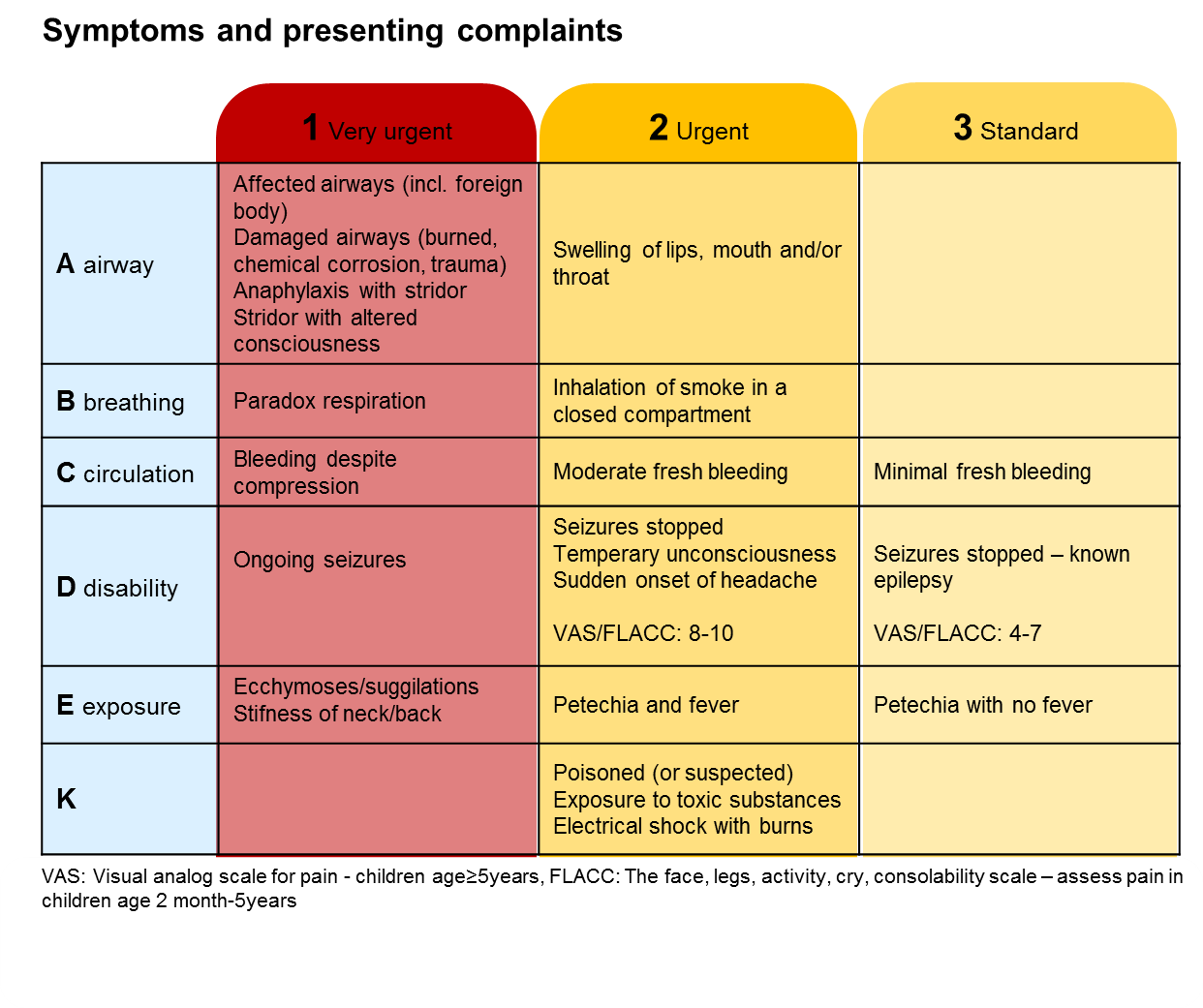
**

**
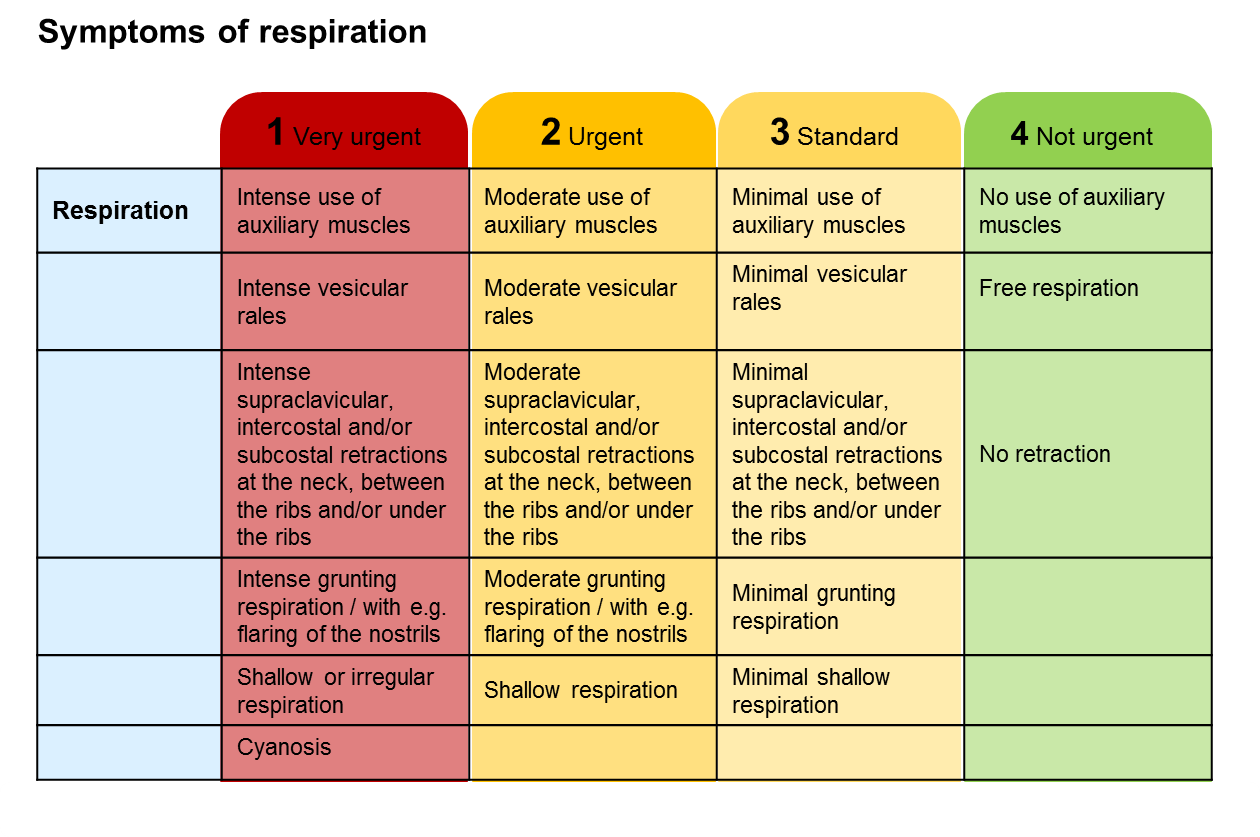
**
